# Characteristics of RSV-Specific Maternal Antibodies in Plasma of Hospitalized, Acute RSV Patients under Three Months of Age

**DOI:** 10.1371/journal.pone.0170877

**Published:** 2017-01-30

**Authors:** Jop Jans, Oliver Wicht, Ivy Widjaja, Inge M. L. Ahout, Ronald de Groot, Teun Guichelaar, Willem Luytjes, Marien I. de Jonge, Cornelis A. M. de Haan, Gerben Ferwerda

**Affiliations:** 1 Laboratory of Pediatric Infectious Diseases, Department of Pediatrics, Radboud Institute for Molecular Life Sciences, Radboud university medical center, Nijmegen, The Netherlands; 2 Centre for Infectious Disease Control, National Institute for Public Health and the Environment (RIVM), Bilthoven, The Netherlands; 3 Virology Division, Department of Infectious Diseases & Immunology, Faculty of Veterinary Medicine, Utrecht University, Utrecht, The Netherlands; Icahn School of Medicine at Mount Sinai, UNITED STATES

## Abstract

Respiratory syncytial virus (RSV) is the leading cause for respiratory illness that requires hospitalization in infancy. High levels of maternal antibodies can protect against RSV infection. However, RSV-infected infants can suffer from severe disease symptoms even in the presence of high levels of RSV-specific antibodies. This study analyzes several serological characteristics to explore potential deficiencies or surpluses of antibodies that could relate to severe disease symptoms. We compare serum antibodies from hospitalized patients who suffered severe symptoms as well as uninfected infants. Disease severity markers were oxygen therapy, tachypnea, oxygen saturation, admission to the intensive care unit and duration of hospitalization. Antibodies against RSV G protein and a prefusion F epitope correlated with *in vitro* neutralization. Avidity of RSV-specific IgG antibodies was lower in RSV-infected infants compared to uninfected controls. Severe disease symptoms were unrelated to RSV-specific IgG antibody titers, avidity of RSV-IgG, virus neutralization capacity or titers against pre- and postfusion F or G protein ectodomains and the prefusion F antigenic site Ø. In conclusion, the detailed serological characterization did not indicate dysfunctional or epitope-skewed composition of serum antibodies in hospitalized RSV-infected infants suffering from severe disease symptoms. It remains unclear, whether specific antibody fractions could diminish disease symptoms.

## Introduction

Human respiratory syncytial virus (RSV) infections are a major burden for infants [1]. The symptoms of RSV infection range from a common cold to severe bronchiolitis and pneumonia. Children below 3 months of age are at risk for developing severe symptoms that require admission to the hospital, whereas the vast majority shows only mild disease [2].

Early in life, the infant’s immune system relies mostly on innate immunity and the presence of maternal, transplacentally transferred immunoglobulin G (matAbs). Consistently, high levels of RSV-specific matAbs in the cord blood are reported to delay the time point of primary RSV infection [3, 4]. Intriguingly, primary infections occur even though high levels of matAbs are present. This raises the question to what extent serum antibodies play a role in infants suffering from severe symptoms during RSV infection and whether antibodies are any different from those of infants with a mild course of disease or uninfected controls. To answer these questions, we investigated whether properties of matAbs such as the level of prefusion F protein-specificity or levels of non-neutralizing antibodies, could relate to severity of disease.

Here, we studied the properties of anti-RSV IgG in plasma of infants that were hospitalized with an acute RSV-infection before 3 months of age. Antibodies observed in a peer group of uninfected individuals represent a base line for: (1) RSV-IgG antibody titers, RSV-IgG avidity, and neutralization capacity, (2) antibody titers against G protein, prefusion F protein, postfusion F protein and (3) antibody titers against prefusion antigenic site Ø and postfusion antigenic site I. The composition of antibodies and/or their combination with properties might represent a signature that is typical for severe RSV disease. We analyzed the antibodies for a correlation with multiple severity parameters, including oxygen therapy, age, respiratory rate, transcutaneous oxygen saturation, admission to an intensive care unit and duration of hospitalization. A better understanding of how antibody properties relate to disease progression is essential to develop safe and effective immunization strategies.

## Materials and Methods

### Study design

Hospitalized children below 3 months of age with PCR-confirmed RSV infections were included during 2011–2013. Within 24 h after admission, a blood sample was taken. Patients with congenital heart or lung disease, immunodeficiency or glucocorticoid use and infants born at a gestational age below 35 weeks were excluded. For primary analyses, patients were classified into two severity groups based on the necessity for oxygen therapy. For secondary analyses, additional disease severity parameters were used to categorize patients: the presence of tachypnea, transcutaneous oxygen saturation (≥93% or <93%), admission to an intensive care unit and duration of hospitalization. The correlation with age was investigated for all analyses to determine whether age would be a potential confounding factor. Infants below 3 months of age requiring surgery for an inguinal hernia repair were included as healthy, uninfected controls. Nasopharyngeal aspirates from all uninfected individuals were RSV negative. The study protocols were approved by the Regional Committee on Research involving Human Subjects Arnhem-Nijmegen (serving as the IRB) and were conducted in accordance with the principles of the Declaration of Helsinki. Written informed consent was obtained from the parents of all children.

### Plasma collection

Coagulation of blood samples was prevented by sodium heparin (BD Vacutainer). After centrifugation at 800 x *g*, the plasma fraction was diluted with an equal volume of phosphate-buffered saline (PBS) and stored at -80°C.

### Culture of virus and neutralization assay

Recombinant RSV-A2 expressing green fluorescent protein (rgRSV) was kindly provided by Dr. Peeples and cultured on HeLa cells as described previously [5]. RSV was purified by ultracentrifugation over a 30% sucrose layer for 1.5h at 20.000 x *g*. RSV was quantified by end point titration assays on HeLa cells.

Recombinant RSV-X expressing green fluorescent protein (RSV-X) has been described earlier as E1-rRSV_X [6]. RSV-X is a more recent clinical isolate of genotype GA2 with 98.5% amino acid sequence identity with RSV-A2 F protein and 86.2% amino acid sequence identity with RSV-A2 G protein. A derivative of recombinant RSV-X that is lacking the G protein was generated by replacing the G protein reading frame by a gene encoding green fluorescent protein (RSV-X-dG) [7]. RSV-X and RSV-X-dG (Dutch genetically modified organism license IG-99-210) were propagated in Vero cells (CCL-81, American Type Culture Collection) using Dulbecco's Modified Eagle Medium (DMEM, Life Technologies) supplemented with 5% FCS and Pen Strep Glutamine (Life Technologies). The cell culture supernatants were concentrated by polyethylene glycol-6000 precipitation and stored with 10% sucrose. Virus titration by end point dilution and plaque reduction neutralization assays were performed as described earlier [6]. The 50% plaque reduction neutralization titers (PRNT) were normalized using a correction factor derived from the intra-assay variation of the WHO513 standard (BEI Resources) and half maximal inhibitory concentrations calculated by fitting a non-linear curve (GraphPad Prism).

### RSV-specific IgG titer and avidity ELISA

RSV-specific IgG concentrations in plasma samples were determined by ELISA. ELISA plates were coated with rgRSV at a concentration of 2 x 10^5^ U/ml in PBS or PBS as negative control. Plates were washed with PBS containing 0.05% Tween-20 and blocked with PBS containing 1% BSA (Sigma-Aldrich). Plasma samples were incubated for 2 h at room temperature (RT). After washing, plates were incubated with alkaline phosphatase (AP)-conjugated anti-human IgG in PBS containing 1% BSA for 2 h (1:10,000; Southern Biotech). Detection of AP activity was performed with 10 mM diethanolamine with 0.5 mM MgCl_2_ using an ELISA plate reader. Optical density at 450 nm wavelength (OD) from PBS-coated wells was subtracted from parallel samples coated with RSV. OD values from serial dilutions of healthy adult pooled plasma ranging 1:10 to 1:10,240 were used as standard to calculate arbitrary units (AU). To determine the IgG avidity of RSV-specific antibodies, wells were treated with sodium thiocyanate (NaSCN, 2 mM) for 10 min to weaken antibody binding and compared to untreated wells [8]. The IgG avidity index was calculated as follows: (RSV-IgG titer without NaSCN (AU) / RSV-IgG titer with NaSCN (AU) *100).

### Recombinant RSV F and G ELISA

Expression constructs, production, and purification of the postfusion F ectodomain (Fwt, called postfusion F protein hereafter), the trimeric, furin cleavage site-mutated, heptad repeat B domain-deleted, prefusion F ectodomain (Flys.ΔHRB-GCN, called prefusion F protein hereafter), and a tetrameric subtype A RSV G ectodomain (residues 64 to 298) have been described previously [9–11]. The antibody response to RSV F or G_A_ was determined using ELISA. NUNC multisorp plates were coated with 25 ng of recombinant protein at 4°C overnight. After washing with PBS containing 0.05% Tween-20, wells were blocked with 3% BSA in PBS containing 0.1% Tween-20 at RT. Two-fold serial dilutions of human patient plasma in blocking buffer were incubated for 1h at RT. After washing, antibodies were labeled with HRP-conjugated goat anti-human IgG (ITK Southern Biotech) for 1h at RT. HRP activity was detected by tetramethylbenzidine substrate (BioFX). The IgG titer for RSV F or G were determined by calculating the corresponding dilution at OD = 1. Antibody response against antigenic site Ø and I was assessed by competition ELISA as previously described [11]. Two-fold serial dilutions of plasma samples or non-labeled monoclonal antibodies (D25 directed at the specific prefusion F antigenic site Ø, and 131-2a directed at a specific postfusion F antigenic site I) in blocking buffer were added and incubated for 1h at RT. After washing, wells were incubated with 0.6 μg/ml biotin-D25 or 0.2 μg/ml biotin-131-2a to enable binding to prefusion F after preincubation with the diluted plasma samples. After 1h at RT, the biotinylated antibodies were labeled with 1 μg/ml HRP-conjugated Streptavidin (Thermo Scientific) and detected by adding tetramethylbenzidine substrate (BioFX) using an ELISA plate reader (EL-808, Biotek). Wells incubated with biotinylated antibodies alone represent uncompeted, positive controls and wells blocking buffer alone served as negative control. Percentage inhibition was calculated for every dilution using the following equation: (OD biotinylated MAb—OD plasma samples) / OD biotinylated MAb X 100. Using a nonlinear 4-parameter fit curve analysis (GraphPad) the inhibition titer for each plasma samples were determined as the plasma dilution that resulted in 25% inhibition of D25 or 15% inhibition of 131-2a binding.

### Statistical analysis

Normality tests were performed on all parameters using the Shapiro-Wilk normality test and showed non-parametric distribution. Comparison between two groups was analyzed by two-tailed Mann-Whitney *U* test and comparison between more than two groups was analyzed by the Kruskall-Wallis test and if significant followed by two-tailed Mann-Whitney *U* test. Spearman correlation test was used for correlation tests. Tests were considered significant if *P* < 0.05. All statistical analyses were done with GraphPad Prism.

## Results

### Study population

Plasma IgG from thirty-three hospitalized RSV patients was compared to eleven uninfected infants. In our primary analysis, the distinction between severe or moderate RSV disease is determined by whether or not patients received oxygen therapy. Medical doctors make the decision to hospitalize patients based on most severe symptoms that indicate oxygen therapy or on moderate symptoms in combination with other indicators, for example frailty or age, but not requiring oxygen. Consistent with more severe disease, the group that received oxygen was hospitalized for a longer period, as compared to RSV-infected infants without oxygen (Table 1). Gender, gestational age, presence of breastfeeding and presence of parental smoking were comparable between all groups (Table 1). Only children below three month of age were considered for this study. The group of uninfected infants was slightly older and had a lower birth weight compared to the RSV-infected groups (Table 1). There was no significant difference in age and duration of symptoms before hospitalization between RSV-infected infants with or without oxygen therapy.

**Table 1:** Study population.

	Healthy (n = 11)	No oxygen (n = 11)	Oxygen (n = 22)	*P* value
**Age [days ± IQR]**	68 [61–80]	58 [36–66]	39.5 [23–70]	<0.05*
**Male gender (%)**	9 (82)	6 (55)	11 (50)	NS
**Gestational age [wk ± IQR]**	37.3 [34.5–39.0]	39.4 [37.3–40.4]	38.6 [36.9–39.9]	NS
**Birth weight [kg ± IQR]**	2.6 [2.13–3.01]	3.16 [2.79–3.62]	3.27 [2.96–3.97]	<0.05^#^
**Breastfeeding (%)**	6 (55)	8 (73)	13 (59)	NS
**Parental smoking (%)**	0 (0)	0 (0)	2 (9)	NS
**Symptomatic [days ± IQR]**	NA	3.0 [3.0–4.0]	3.5 [2.0–5.0]	NS
**Duration hospitalization [days ± IQR]**	NA	3.0 [2.0–6.0]	10.0 [8.0–11.3]	<0.001^+^

Plasma from infants with RSV infections and healthy controls were included and divided by disease severity based on oxygen therapy. Values are depicted as medians ± interquartile range (IQR) or number with percentage for categorical data. Testing was performed with Kruskall-Wallis and if significant followed by two-tailed Mann Whitney *U* test.

* Healthy versus no oxygen p<0.05, healthy versus oxygen p<0.01, oxygen versus no oxygen NS.

^#^ Healthy versus no oxygen p<0.05, healthy versus oxygen p<0.05, oxygen versus no oxygen NS.

^+^ oxygen versus no oxygen <0.001.

### Level and avidity of RSV-specific IgG in infant sera

**Fig 1:**
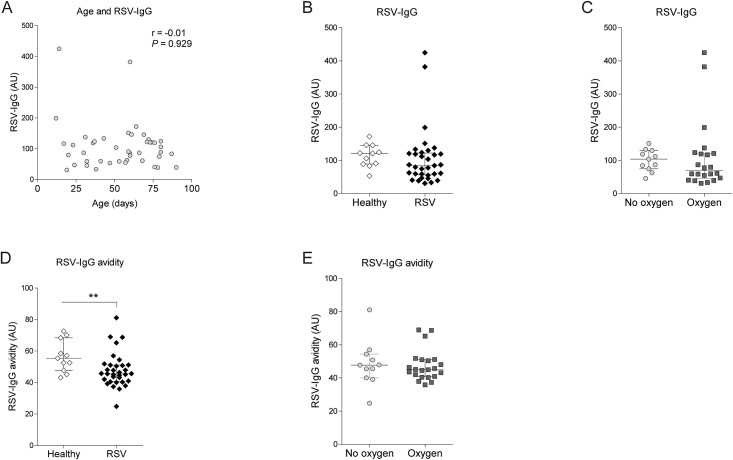
RSV-specific IgG titer and RSV-IgG avidity do not correlate with disease severity. RSV-specific IgG levels in plasma of infants was determined by ELISA using virus particles. RSV-IgG avidity was assessed by supplementing NaSCN during ELISA. (A) RSV-specific IgG levels were displayed versus age. (B-C) Median RSV-specific IgG levels (± IQR) were compared between healthy and RSV-infected infants as well as between RSV patients with and without oxygen therapy. (D-E) Median (± IQR) avidity of RSV-IgG in healthy infants was compared to RSV-infected infants or between RSV patients with or without oxygen therapy. Associations were assessed by Spearman correlation test. Statistical analyses employed Mann Whitney U test. (**P<0.01).

Age is correlated with a decay of matAbs and young age is a risk factor for severe RSV disease [12]. In this study, we observed no correlation between age at RSV infection and the levels of RSV-specific IgG (Fig 1A). Moreover, there was no significant difference in RSV-IgG levels between RSV patients and healthy children (Fig 1B). Next, we asked how antibody levels relate to severity of disease. We observed similar levels of RSV-specific IgG in RSV-infected children with or without oxygen therapy (Fig 1C). In addition, similar serum RSV-IgG levels were found when comparing other measures of severity (Table 2). The presence of low-avidity RSV-specific IgG enhances disease symptoms in animal models [13]. RSV-specific IgG in plasma from RSV-infected infants had a lower RSV-IgG avidity compared to healthy controls, respectively 45 [41–51] versus 55 [48–68] (Fig 1D). However, no difference in RSV-IgG avidity was observed when categorizing RSV patients based on oxygen therapy or other disease severity measures (Fig 1E and Table 2).

**Table 2:** Antibody properties do not correlate with disease severity parameters.

	**RSV-IgG (AU) [IQR]**	***P* value**	**RSV-IgG avidity (AU) [IQR]**	***P* value**	**Neutralization RSV-X (PRNT log2) [IQR)**	***P* value**
**Tachypnea (absent vs present)**	112 [68–138] vs 79 [52–121]	0.299	47 [42–51] vs 45 [40–51]	0.828	9.4 [7.9–10.4] vs 9.1 [8.3–9.7]	0.560
**Saturation (≥93% vs <93%)**	87 [55–130] vs 77 [60–122]	1.000	46 [40–52] vs 46 [43–51]	0.782	9.1 [8.2–9.4] vs 9.5 [7.9–9.9]	0.434
**ICU admission (absent vs present)**	87 [60–127] vs 70 [40–119]	0.303	48 [41–51] vs 45 [40–50]	0.359	9.1 [8.0–9.5] vs 9.6 [8.9–10.0]	0.184
**Duration hospitalization (correlation)**	No correlation	0.101	No correlation	0.108	No correlation	0.918
**Age (correlation)**	No correlation	0.929	No correlation	0.070	No correlation	0.089
	**Neutralization RSV-X-dG (PRNT log2) [IQR]**	***P* value**	**G protein IgG (AU) [IQR]**	***P* value**	**Prefusion F protein IgG (AU) [IQR]**	***P* value**
**Tachypnea (absent vs present)**	7.9 [6.8–10.0] vs 7.8 [6.7–9.0]	0.469	216 [151–383] vs 209 [127–478]	0.754	179 [119–263] vs 215 [110–365]	0.717
**Saturation (≥93% vs <93%)**	7.6 [6.7–9.0] vs 8.0 [6.8–9.1]	0.490	161 [122–272] vs 261 [186–572]	0.072	202 [111–303] vs 225 [138–418]	0.357
**ICU admission (absent vs present)**	7.6 [6.7–8.7] vs 8.5 [7.1–9.1]	0.303	200 [134–414] vs 207 [80–416]	0.667	202 [138–356] vs 262 [103–430]	0.955
**Duration hospitalization (correlation)**	No correlation	0.884	No correlation	0.116	No correlation	0.698
**Age (correlation)**	No correlation	0.129	No correlation	0.409	No correlation	0.109
	**Postfusion F protein IgG (AU) [IQR]**	***P* value**	**D25 inhibition (25%inhibition) [IQR]**	***P* value**	**131-2A inhibition (15% inhibition) [IQR]**	***P* value**
**Tachypnea (absent vs present)**	647 [236–888] vs 529 [245–894]	0.828	5.1 [4.2–6.0] vs 5.4 [4.7–6.4]	0.334	5.0 [4.6–5.7] vs 5.4 [5.2–5.5]	0.328
**Saturation (≥93% vs <93%)**	590 [296–1415] vs 546 [242–770]	0.645	5.1 [4.7–5.9] vs 5.4 [4.7–6.8]	0.685	5.4 [5.0–6.0] vs 5.2 [4.6–5.5]	0.397
**ICU admission (absent vs present)**	590 [238–1078] vs 388 [262–743]	0.640	5.5 [4.7–6.2] vs 5.3 [4.7–7.0]	0.829	5.4 [5.0–5.8] vs 5.4 [4.7–5.8]	0.762
**Duration hospitalization (correlation)**	No correlation	0.101	No correlation	0.504	No correlation	0.980
**Age (correlation)**	No correlation	0.450	No correlation	0.268	No correlation	0.243

Antibody properties were assessed for correlation with multiple disease severity markers. Values are depicted as medians ± IQR. Statistical analyses employed Mann Whitney *U* test. Testing for correlation was performed with Spearman correlation test. *P* values are depicted.

### RSV neutralization capacity of infant sera

**Fig 2:**
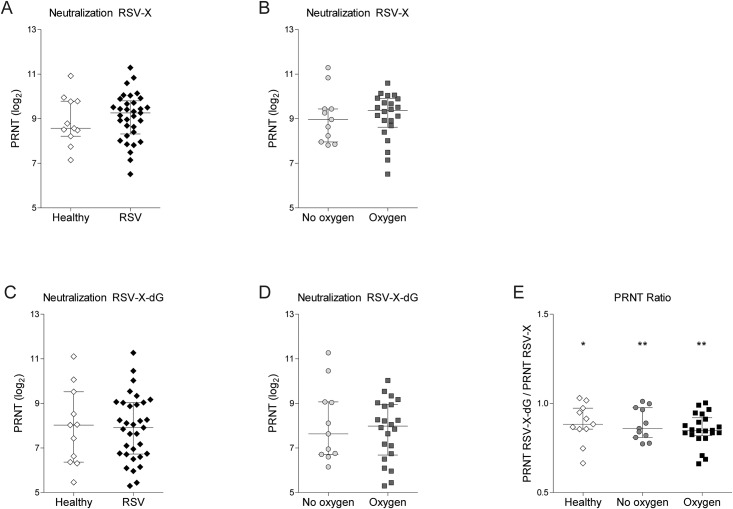
RSV neutralization does not correlate with severity of disease. 50% plaque reduction neutralization titers (PRNT) were determined against RSV-X and RSV-X-dG, the latter is lacking the G protein. (A-B) Median (± IQR) PRNT against RSV-X were compared between healthy and RSV-infected infants as well as between RSV patients with and without oxygen therapy. (C-D) Median (± IQR) PRNT against RSV-X-dG were compared between healthy and RSV-infected infants as well as between RSV patients with or without oxygen therapy. (E) The PRNT against RSV-X was divided the PRTN against RSV-X-dG, so that values below 1.0 indicate a contribution of anti-G antibodies to the neutralization. The median (± IQR) ratio was compared between plasma from healthy infants and RSV patients with or without oxygen therapy and all were significantly different from 1.0. Statistical analyses employed Mann Whitney *U* test for comparison between two groups and Kruskall-Wallis for comparison between more than two groups. (*P<0.05, **P<0.01).

Next, we investigated whether the RSV neutralization capacity *in vitro* relates to RSV infection or severity of disease. We determined the capacity of patient plasma to neutralize infection by RSV-X and the G protein-deficient RSV-X-dG. Plasma from healthy controls and RSV-infected infants neutralized RSV-X and RSV-X-dG to a similar extent (Fig 2A and 2C). Also, the median PRNT against both viruses was similar between RSV-infected infants with or without oxygen therapy (Fig 2B and 2D). In addition, no difference in neutralization of RSV-X and RSV-X-dG was observed when categorizing RSV-infected infants based on other disease severity parameters (Table 2). Furthermore, we analyzed whether the contribution of anti-G antibodies to neutralization was different between infant groups. To that end, ratios were calculated from the PRNT using RSV-X and RSV-X-dG. Average PRNT ratios of 0.9 indicated that 10% of the neutralizing activity could be contributed by anti-G antibodies (Fig 2E). These average PRNT ratios were significantly different from 1, which would occur if RSV-X and RSV-X-dG were neutralized equally well. A similar PRNT ratio of 0.9 was observed when comparing RSV-infected infants based on oxygen therapy and healthy infants (Fig 2E). The functionality of this assay was verified by measuring the ratio of RSV-X and RSV-X-dG neutralization by palivizumab, a monoclonal antibody that neutralizes RSV by binding only to the F protein. Thus, palivizumab is independent of the absence or presence of the G protein. Correspondingly, the ratio of PRNT for RSV-X and RSV-X-dG for palivizumab was 1.01.

### RSV-specific IgG against viral surface antigens

**Fig 3:**
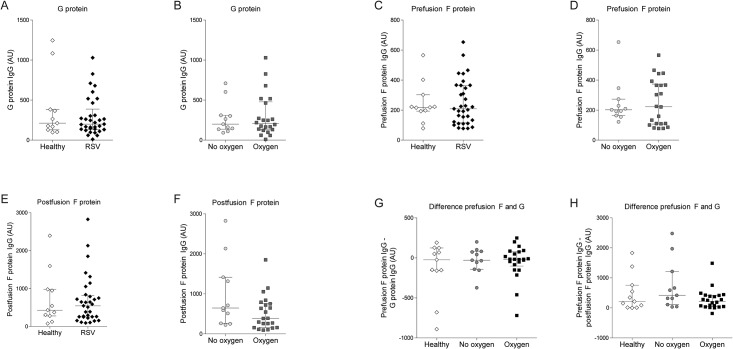
RSV-specific IgG against individual RSV glycoproteins do not correlate with disease severity. RSV glycoprotein-specific antibody levels were determined by ELISA against recombinant, soluble ectodomains. Median IgG levels (± IQR) were compared between healthy and RSV-infected infants as well as between RSV patients with and without oxygen therapy. Glycoproteins used for ELISA coating were (A-B) RSV G protein, (C-D) prefusion F protein, and (E-F) postfusion F protein. (G-H) The relative abundance of antibodies against prefusion F compared to G protein as well as pre- compared to postfusion F protein were categorized assessed by subtracting their levels. The median (± IQR) difference was compared between plasma from healthy infants and RSV patients with or without oxygen therapy. No significant differences were observed by Mann Whitney *U* test for comparison between two groups and Kruskall-Wallis for comparison between more than two groups.

An excess of anti-G antibodies have been linked with the failure of the formalin-inactivated RSV vaccine in the 1960s and enhanced disease in mice [14, 15]. Also, antibodies specific for the postfusion F protein seem not to contribute to RSV neutralization [16]. We tested whether antibodies against individual antigens would correlate with disease severity. The levels of antigen-specific IgG present in plasma were determined using soluble, recombinant G protein, pre- and postfusion F protein ectodomains in ELISA. Median antibody levels against G protein were comparable in plasma derived from healthy infants and RSV infections (Fig 3A). Similarly, we did not find a difference of antibody levels against pre- or postfusion F proteins between healthy infants and RSV-infected infants (Fig 3C and 3E). The same was true for median antibody levels when categorizing RSV infections based on oxygen therapy or other disease severity parameters (Fig 3B, 3D, 3F and Table 2). To investigate whether the balance between different antibodies is associated with disease severity, we analyzed the difference between prefusion F and G-specific antibodies as well as pre- and postfusion F–specific antibodies by subtracting antibody levels against G protein or postfusion F protein from those against prefusion F protein. The relative abundance of RSV-antigens was comparable in all groups (Fig 3G and 3H), thus no symptom-related antibody composition was identified.

### RSV-specific IgG against a prefusion F epitope

**Fig 4:**
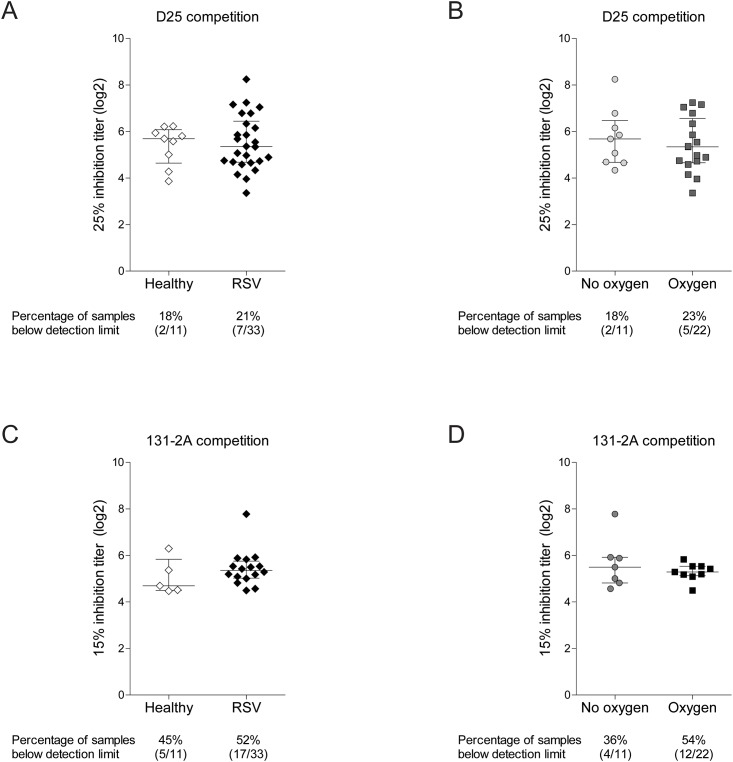
RSV-specific IgG against F protein antigenic sites Ø and I not correlate with disease severity. The abundance of antibodies in human infant plasma that bind to the prefusion F protein antigenic site Ø or the postfusion F protein antigenic site I was determined by competition with site-specific monoclonal antibodies in ELISA. (A-B) Median (± IQR) IgG titer that blocks 25% binding of D25 (site Ø) were compared between healthy and RSV-infected infants as well as between RSV patients with and without oxygen therapy. (C-D) Median (± IQR) IgG titer that blocks 15% binding of 131-2A (site I) were compared between healthy and RSV-infected infants as well as between RSV patients with and without oxygen therapy. No significant differences were observed by Mann Whitney *U* test.

**Fig 5:**
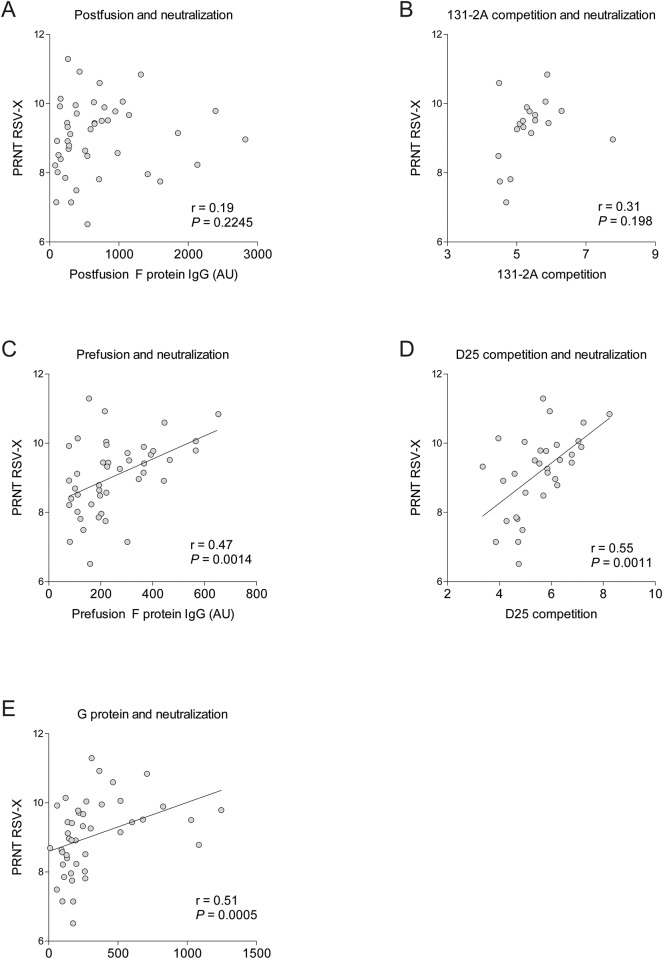
RSV-specific IgG against prefusion F epitope and G protein correlate with neutralization. Neutralization titers against RSV-X (PRNT) were compared to IgG levels against (A) postfusion F protein, (B) site I (131-2A competition titer), (C) prefusion F protein, (D) site Ø (D25 competition titer) and (E) G protein. Testing for correlation was performed with Spearman correlation test.

Antibodies against prefusion F protein have received much attention recently, because they correlate best with RSV neutralization *in vitro*. We refined our analysis to detect antibodies against epitopes that discriminate between pre- and postfusion F [16, 17]. Sera from healthy and RSV-infected infants were titrated to compete with the monoclonal antibody D25 for prefusion-specific antigenic site Ø or with the monoclonal antibody 131-2a for the postfusion-specific epitope I [9, 11, 16, 18]. The median competition titers were similar between healthy infants and RSV-infected infants for D25 (Fig 4A) and for 131-2a (Fig 4C). We observed no difference in D25 competition or 131-2A competition when categorizing RSV infections based on oxygen therapy (Fig 4B, 4D and Table 2). A previous study showed that prefusion F protein-specific antibodies determine the neutralization activity of seroconverted individuals older than 6 years of age [16]. To assess whether this is represented in our measurements of RSV-specific matAbs, we considered the relationship between antigen- and epitope-specific antibody titers and neutralizing capacity (PRNT). Antibodies against postfusion F and 131-2a competition titers represent a negative control and were not associated with neutralization (Fig 5A and 5B). Prefusion F-specific antibodies and D25 competition titers correlated with PRNT (Fig 5C and 5D). Furthermore, antibody titers against G protein also correlated positively with PRNT (Fig 5E).

## Discussion

Infants encounter primary RSV infections even though matAbs are present in their blood. We investigated whether individual serologic properties of antibodies or any signature combinations thereof would be related to the observed disease symptoms. Our results indicate that (i) disease severity is not associated with differences in RSV-specific IgG titers, RSV-IgG avidity, or virus neutralization and (ii) plasma titers of RSV-specific IgG against the G protein and the prefusion F protein correlate with neutralizing capacity. In addition, (iii) IgG titers directed against the G protein, prefusion F protein, postfusion F protein, F-antigenic site Ø and site I, or any combination of thereof could not be associated with severity of symptoms.

Plasma from infants below 3 months of age was selected because of the high incidence of severe RSV infection even though high levels of matAbs are present in this group. Indeed, we found a median RSV neutralization titers of about 1:512 for all infants (Fig 2), which is similar to what is reported for cord blood in some studies [19, 20]. We omitted age as a confounder for different severity of symptoms, as the age of RSV-infected infants without oxygen therapy was comparable to that of oxygen-treated children. A potential limitation of our study is the difficulty to separate the presence of matAbs from endogenous RSV-induced antibodies of the infant during infection. With a median onset of disease of three days and the short time between symptoms and hospitalization, production of endogenous RSV-IgG is improbable. Moreover, as Dutch newborns in general suffer their first respiratory infection after 3–7 months of age and only 11% of respiratory infections within the first year of life are caused by RSV, infants in our study most likely experienced a primary RSV infection [21]. Therefore, it is plausible that we detect matAbs [22]. Our plasma selection facilitates the comparison of antibodies from hospitalized patients with symptoms ranging from moderate to severe. As this is not a prospective study, the ideal control group of RSV-infected children with mild symptoms was not available, because blood of these children is not routinely sampled. A common practice in RSV research is to use age-matched infants as control. One assumes that this group is not protected and if healthy infants become RSV infected, a vast majority of 98–99% would develop only mild symptoms [23]. We too, substituted the group with mild disease by the analysis of antibodies from uninfected infants. This has no significant impact on our findings and conclusions. The sample size of our control group is relatively small and definitive conclusions in the context of correlation with infection warrant future studies. Nevertheless, the median levels of antibody properties are in close proximity with the median levels of RSV-infected infants. Therefore, it seems unlikely that an addition of more control samples would significantly affect our interpretation. Future studies may include mild infections or prospectively follow healthy infants until RSV infections occurs to investigate the protective effect of RSV-specific antibodies.

High titers of IgG matAbs against RSV have been inversely associated with prevention of hospitalization for RSV disease [24], whereas mucosal IgA reduces the propensity to become infected with RSV [25]. We did not study IgA-mediated prevention of infection, because RSV-IgA is not transplacentally transferred during gestation. We specifically investigated IgG antibodies during an acute infection and observed no correlation between RSV-IgG titer or RSV neutralization and severity of symptoms. Differences in the design of studies investigating prevention of RSV infection versus prevention of severe disease symptoms in hospitalized infants may explain the different conclusion. However, our data is in line with some studies that describe that hospitalization during RSV disease is not correlated to matAb levels [22, 26]. Furthermore, we found RSV-IgG avidity from uninfected infants was significantly higher than from RSV patients, but was not associated with disease severity. Previous studies in mice suggest that non-neutralizing and low affinity antibodies could contribute to the enhanced pathology [13, 14, 27]. The biological relevance of the small but significant differences in RSV-IgG avidity between uninfected infants and infants with RSV infection should be carefully interpreted, because the threshold for antibody avidity to be efficacious during RSV infections is currently unknown. These results should be validated with a prospective study to determine the association of avidity and the protection against severe RSV infections in infants.

As global antibody properties such as titers and neutralization capacity did not correlate with severity of disease, we analyzed whether this was also the case for antibodies against specific RSV antigens. Anti-G protein antibodies have been suggested to facilitate immunopathology in children [14, 27]. In contrast, RSV neutralizing antibodies are usually directed against prefusion F and can be administered as prophylaxis [16, 28]. By comparing the PRNT of patient plasma against full length RSV-X and RSV-X-dG, which lacks the G gene, we observed that anti-G protein antibodies contribute to neutralization *in vitro*. This is supported by a positive relationship between anti-G protein antibody titers and PRNT. However, the median anti-G antibody titers were similar across all infant sera, regardless of disease and severity. Our serology using RSV A-derived materials does not account for potential impact of the RSV genotype on disease. We believe this is not affecting the interpretation of our results, because only a single serotype for RSV exists. Plasma antibodies from human infants prior to RSV infection, i.e. maternal antibodies, generally show very similar activity against major RSV subtypes regarding the conserved F protein but also the more variable G protein [29]. Moreover, different RSV genotypes have not been convincingly or continuously linked to the epidemiology of RSV outbreaks. We next asked whether the ratios between antibodies against G, pre- and postfusion F would represent a specific signature in severely sick infants compared to mildly sick children and controls. We found that the balance between prefusion F and other antibodies was similar in all infants and independent of their disease severity.

Recent data suggests that especially antibodies targeting the prefusion F protein epitope Ø efficiently neutralize RSV [30, 31]. To determine whether infants suffering from severe disease symptoms have different epitope Ø binding antibodies compared to controls, we analyzed patient plasma from all groups in a competition assay with D25 and 131-2A. As we did not find any difference, we argue that sera from mildly sick RSV patients have average epitope Ø-specific antibody titers just like children with severe symptoms. We did observe an association between recognition of the D25 epitope and the PRNT of matAbs, like previously shown in seroconverted individuals above 6 years of age [16]. We tested for this prefusion F specific antibody because it is the focus of current therapeutic developments. We note that there are more prefusion epitopes that could theoretically correlate with disease.

Apart from interacting with viral antigens on virions, antibodies also have Fc region-mediated immunological functions during natural infection. For example, antibodies form immune complexes that can reshape the cytokine and chemokine response of human immune cells [32, 33] or facilitate complement deposition. We did not study antibody effector functions that could relate to severity of disease.

In conclusion, this study investigated the properties of naturally occurring RSV-specific matAbs hoping to provide clues about a specific signature of antibodies that is needed to prevent severe RSV infections. Our detailed characterization did not find any difference in matAbs between infants with severe RSV disease symptoms, controls with mild RSV disease and uninfected infants. This means that the matAb properties we studied do not indicate whether a child would be protected from severe symptoms. It will be very important to find such properties or signatures in order to design effective vaccination strategies in the future.

## Supporting Information

S1 Table Supporting information depicting all individual data. (XLS)

## References

[1] Nair H, Nokes DJ, Gessner BD, Dherani M, Madhi SA, Singleton RJ, et al Global burden of acute lower respiratory infections due to respiratory syncytial virus in young children: a systematic review and meta-analysis. Lancet. 2010;375(9725):1545–55. 10.1016/S0140-6736(10)60206-1 20399493 PMC2864404

[2] Zorc JJ, Hall CB. Bronchiolitis: recent evidence on diagnosis and management. Pediatrics. 2010;125(2):342–9. 10.1542/peds.2009-2092 20100768

[3] Chu HY, Steinhoff MC, Magaret A, Zaman K, Roy E, Langdon G, et al Respiratory syncytial virus transplacental antibody transfer and kinetics in mother-infant pairs in Bangladesh. The Journal of infectious diseases. 2014;210(10):1582–9. Epub 2014/06/07. 10.1093/infdis/jiu316 24903663 PMC4334795

[4] Glezen WP, Paredes A, Allison JE, Taber LH, Frank AL. Risk of respiratory syncytial virus infection for infants from low-income families in relationship to age, sex, ethnic group, and maternal antibody level. J Pediatr. 1981;98(5):708–15. 7229749 10.1016/s0022-3476(81)80829-3

[5] Vissers M, Hartman Y, Groh L, de Jong DJ, de Jonge MI, Ferwerda G. Recognition of Streptococcus pneumoniae and muramyl dipeptide by NOD2 results in potent induction of MMP-9, which can be controlled by lipopolysaccharide stimulation. Infect Immun. 2014;82(12):4952–8. 10.1128/IAI.02150-14 25183734 PMC4249303

[6] van Remmerden Y, Xu F, van Eldik M, Heldens JG, Huisman W, Widjojoatmodjo MN. An improved respiratory syncytial virus neutralization assay based on the detection of green fluorescent protein expression and automated plaque counting. Virol J. 2012;9:253 10.1186/1743-422X-9-253 23114196 PMC3514128

[7] Widjojoatmodjo MN, Boes J, van Bers M, van Remmerden Y, Roholl PJ, Luytjes W. A highly attenuated recombinant human respiratory syncytial virus lacking the G protein induces long-lasting protection in cotton rats. Virol J. 2010;7:114 10.1186/1743-422X-7-114 20525213 PMC2887800

[8] Vermont CL, van Dijken HH, van Limpt CJ, de Groot R, van Alphen L, van Den Dobbelsteen GP. Antibody avidity and immunoglobulin G isotype distribution following immunization with a monovalent meningococcal B outer membrane vesicle vaccine. Infection and immunity. 2002;70(2):584–90. 10.1128/IAI.70.2.584-590.2002 11796586 PMC127718

[9] Widjaja I, Rigter A, Jacobino S, van Kuppeveld FJ, Leenhouts K, Palomo C, et al Recombinant Soluble Respiratory Syncytial Virus F Protein That Lacks Heptad Repeat B, Contains a GCN4 Trimerization Motif and Is Not Cleaved Displays Prefusion-Like Characteristics. PloS one. 2015;10(6):e0130829 10.1371/journal.pone.0130829 26107504 PMC4481108

[10] Rigter A, Widjaja I, Versantvoort H, Coenjaerts FE, van Roosmalen M, Leenhouts K, et al A protective and safe intranasal RSV vaccine based on a recombinant prefusion-like form of the F protein bound to bacterium-like particles. PLoS One. 2013;8(8):e71072 10.1371/journal.pone.0071072 23951084 PMC3741363

[11] Widjaja I, Wicht O, Luytjes W, Leenhouts K, Rottier PJ, van Kuppeveld FJ, et al Characterization of Epitope-Specific Anti-Respiratory Syncytial Virus (Anti-RSV) Antibody Responses after Natural Infection and after Vaccination with Formalin-Inactivated RSV. J Virol. 2016;90(13):5965–77. 10.1128/JVI.00235-16 27099320 PMC4907225

[12] Wright PF, Gruber WC, Peters M, Reed G, Zhu Y, Robinson F, et al Illness severity, viral shedding, and antibody responses in infants hospitalized with bronchiolitis caused by respiratory syncytial virus. The Journal of infectious diseases. 2002;185(8):1011–8. 10.1086/339822 11930309

[13] Delgado MF, Coviello S, Monsalvo AC, Melendi GA, Hernandez JZ, Batalle JP, et al Lack of antibody affinity maturation due to poor Toll-like receptor stimulation leads to enhanced respiratory syncytial virus disease. Nat Med. 2009;15(1):34–41. 10.1038/nm.1894 19079256 PMC2987729

[14] Murphy BR, Prince GA, Walsh EE, Kim HW, Parrott RH, Hemming VG, et al Dissociation between serum neutralizing and glycoprotein antibody responses of infants and children who received inactivated respiratory syncytial virus vaccine. J Clin Microbiol. 1986;24(2):197–202. 3755730 10.1128/jcm.24.2.197-202.1986 PMC268874

[15] Hancock GE, Speelman DJ, Heers K, Bortell E, Smith J, Cosco C. Generation of atypical pulmonary inflammatory responses in BALB/c mice after immunization with the native attachment (G) glycoprotein of respiratory syncytial virus. J Virol. 1996;70(11):7783–91. 8892899 10.1128/jvi.70.11.7783-7791.1996 PMC190848

[16] Ngwuta JO, Chen M, Modjarrad K, Joyce MG, Kanekiyo M, Kumar A, et al Prefusion F-specific antibodies determine the magnitude of RSV neutralizing activity in human sera. Sci Transl Med. 2015;7(309):309ra162. 10.1126/scitranslmed.aac4241 PMC4672383 26468324

[17] Arbiza J, Taylor G, Lopez JA, Furze J, Wyld S, Whyte P, et al Characterization of two antigenic sites recognized by neutralizing monoclonal antibodies directed against the fusion glycoprotein of human respiratory syncytial virus. J Gen Virol. 1992;73 (Pt 9):2225–34. 1383404 10.1099/0022-1317-73-9-2225

[18] McLellan JS, Yang Y, Graham BS, Kwong PD. Structure of respiratory syncytial virus fusion glycoprotein in the postfusion conformation reveals preservation of neutralizing epitopes. J Virol. 2011;85(15):7788–96. 10.1128/JVI.00555-11 21613394 PMC3147929

[19] Nyiro JU, Sande C, Mutunga M, Kiyuka PK, Munywoki PK, Scott JA, et al Quantifying maternally derived respiratory syncytial virus specific neutralising antibodies in a birth cohort from coastal Kenya. Vaccine. 2015;33(15):1797–801. Epub 2015/03/01. 10.1016/j.vaccine.2015.02.039 25725445 PMC4376380

[20] Atwell JE, Thumar B, Robinson LJ, Tobby R, Yambo P, Ome-Kaius M, et al Impact of Placental Malaria and Hypergammaglobulinemia on Transplacental Transfer of Respiratory Syncytial Virus Antibody in Papua New Guinea. The Journal of infectious diseases. 2016;213(3):423–31. Epub 2015/08/05. 10.1093/infdis/jiv401 26238686 PMC4704666

[21] van der Zalm MM, Uiterwaal CS, Wilbrink B, de Jong BM, Verheij TJ, Kimpen JL, et al Respiratory pathogens in respiratory tract illnesses during the first year of life: a birth cohort study. The Pediatric infectious disease journal. 2009;28(6):472–6. Epub 2009/06/09. 19504730 10.1097/inf.0b013e318195e26e

[22] Freitas GR, Silva DA, Yokosawa J, Paula NT, Costa LF, Carneiro BM, et al Antibody response and avidity of respiratory syncytial virus-specific total IgG, IgG1, and IgG3 in young children. J Med Virol. 2011;83(10):1826–33. 10.1002/jmv.22134 21837801

[23] Bont L, Checchia PA, Fauroux B, Figueras-Aloy J, Manzoni P, Paes B, et al Defining the Epidemiology and Burden of Severe Respiratory Syncytial Virus Infection Among Infants and Children in Western Countries. Infectious diseases and therapy. 2016;5(3):271–98. Epub 2016/08/03. 10.1007/s40121-016-0123-0 27480325 PMC5019979

[24] Stensballe LG, Ravn H, Kristensen K, Agerskov K, Meakins T, Aaby P, et al Respiratory syncytial virus neutralizing antibodies in cord blood, respiratory syncytial virus hospitalization, and recurrent wheeze. J Allergy Clin Immunol. 2009;123(2):398–403. 10.1016/j.jaci.2008.10.043 19101023

[25] Habibi MS, Jozwik A, Makris S, Dunning J, Paras A, DeVincenzo JP, et al Impaired Antibody-mediated Protection and Defective IgA B-Cell Memory in Experimental Infection of Adults with Respiratory Syncytial Virus. Am J Respir Crit Care Med. 2015;191(9):1040–9. 10.1164/rccm.201412-2256OC 25730467 PMC4435460

[26] Eick A, Karron R, Shaw J, Thumar B, Reid R, Santosham M, et al The role of neutralizing antibodies in protection of American Indian infants against respiratory syncytial virus disease. The Pediatric infectious disease journal. 2008;27(3):207–12. 10.1097/INF.0b013e31815ac585 18277934

[27] Kapikian AZ, Mitchell RH, Chanock RM, Shvedoff RA, Stewart CE. An epidemiologic study of altered clinical reactivity to respiratory syncytial (RS) virus infection in children previously vaccinated with an inactivated RS virus vaccine. Am J Epidemiol. 1969;89(4):405–21. 4305197 10.1093/oxfordjournals.aje.a120954

[28] Kwakkenbos MJ, Diehl SA, Yasuda E, Bakker AQ, van Geelen CM, Lukens MV, et al Generation of stable monoclonal antibody-producing B cell receptor-positive human memory B cells by genetic programming. Nat Med. 2010;16(1):123–8. 10.1038/nm.2071 20023635 PMC2861345

[29] Shinoff JJ, O'Brien KL, Thumar B, Shaw JB, Reid R, Hua W, et al Young infants can develop protective levels of neutralizing antibody after infection with respiratory syncytial virus. The Journal of infectious diseases. 2008;198(7):1007–15. 10.1086/591460 18702606

[30] Krarup A, Truan D, Furmanova-Hollenstein P, Bogaert L, Bouchier P, Bisschop IJ, et al A highly stable prefusion RSV F vaccine derived from structural analysis of the fusion mechanism. Nature communications. 2015;6:8143. 10.1038/ncomms9143 PMC4569726 26333350

[31] McGinnes Cullen L, Schmidt MR, Kenward SA, Woodland RT, Morrison TG. Murine immune responses to virus-like particle-associated pre- and postfusion forms of the respiratory syncytial virus F protein. J Virol. 2015;89(13):6835–47. 10.1128/JVI.00384-15 25903340 PMC4468467

[32] Polack FP, Teng MN, Collins PL, Prince GA, Exner M, Regele H, et al A role for immune complexes in enhanced respiratory syncytial virus disease. J Exp Med. 2002;196(6):859–65. 10.1084/jem.20020781 12235218 PMC2194058

[33] Gomez RS, Ramirez BA, Cespedes PF, Cautivo KM, Riquelme SA, Prado CE, et al Contribution of Fcgamma receptors to human respiratory syncytial virus pathogenesis and the impairment of T-cell activation by dendritic cells. Immunology. 2016;147(1):55–72. 10.1111/imm.12541 26451966 PMC4693880

